# Characterizing light-dark cycles in the Neonatal Intensive Care Unit: a retrospective observational study

**DOI:** 10.3389/fphys.2023.1217660

**Published:** 2023-08-16

**Authors:** Isabelle A. Van der Linden, Esther M. Hazelhoff, Eline R. De Groot, Daniel C. Vijlbrief, Luc J. M. Schlangen, Yvonne A. W. De Kort, Marijn J. Vermeulen, Demy Van Gilst, Jeroen Dudink, Laura Kervezee

**Affiliations:** ^1^ Department of Neonatology, Wilhelmina Children’s Hospital, University Medical Center Utrecht, Utrecht, Netherlands; ^2^ Laboratory for Neurophysiology, Department of Cellular and Chemical Biology, Leiden University Medical Center, Leiden, Netherlands; ^3^ Department of Industrial Engineering and Innovation Sciences, Eindhoven University of Technology, Eindhoven, Netherlands; ^4^ Department of Neonatal and Pediatric Intensive Care, Division of Neonatology, Erasmus MC—Sophia Children’s Hospital, Rotterdam, Netherlands

**Keywords:** light, NICU, preterm birth, light-dark cycles, phototherapy, chronobiology, neonatology, circadian rhythms

## Abstract

**Objectives:** To characterize bedside 24-h patterns in light exposure in the Neonatal Intensive Care Unit (NICU) and to explore the environmental and individual patient characteristics that influence these patterns in this clinical setting.

**Methods:** We conducted a retrospective cohort study that included 79 very preterm infants who stayed in an incubator with a built-in light sensor. Bedside light exposure was measured continuously (one value per minute). Based on these data, various metrics (including relative amplitude, intradaily variability, and interdaily stability) were calculated to characterize the 24-h patterns of light exposure. Next, we determined the association between these metrics and various environmental and individual patient characteristics.

**Results:** A 24-h light-dark cycle was apparent in the NICU with significant differences in light exposure between the three nurse shifts (*p* < 0.001), with the highest values in the morning and the lowest values at night. Light exposure was generally low, with illuminances rarely surpassing 75 lux, and highly variable between patients and across days within a single patient. Furthermore, the season of birth and phototherapy had a significant effect on 24-h light-dark cycles, whereas no effect of bed location and illness severity were observed.

**Conclusion:** Even without an official lighting regime set, a 24-h light-dark cycle was observed in the NICU. Various rhythmicity metrics can be used to characterize 24-h light-dark cycles in a clinical setting and to study the relationship between light patterns and health outcomes.

## 1 Introduction

Very preterm infants (i.e., babies born before 32 weeks of gestation) leave the controlled intrauterine environment prematurely and–if available–are then admitted to a Neonatal Intensive Care Unit (NICU). Over the last decades, survival rates of these babies have improved considerably (Ancel et al., 2015). Therefore, it is becoming increasingly important to focus on optimizing the NICU environment to limit complications of prematurity, in order to improve clinical outcomes and quality of life (World Health Organization et al., 2012).

The NICU is an environment which usually lacks clear daily rhythmicity (McKenna and Reiss, 2018; Hazelhoff et al., 2021). Examples of the current practice include exposure to constant light levels over the entire day, continuous administration of nutrition, and around-the-clock care activities without taking into account the infants’ sleep-wake cycle (McKenna and Reiss, 2018; Hazelhoff et al., 2021). The current standard of lighting in the NICU is ambient lighting levels that are adjustable through a range of 10 lux (comparable to twilight) to a maximum of 600 lux (comparable to regular office lighting), with no recommendation on the timing or spectrum of light (White, 2020). All these environmental factors without a clear 24-h rhythm are in contrast to the rhythmic environment that a fetus is exposed to inside the womb. Here, the fetus receives timing cues from the mother through daily physiological rhythms in hormones, body temperature, physical activity and/or circulating nutrients (Serón-Ferré et al., 2012; Bates and Herzog, 2020). These 24-h rhythms are driven by the circadian timing system of the mother and are entrained to the external light-dark cycle by her central circadian clock located in the suprachiasmatic nuclei (SCN) (Sollars and Pickard, 2015).

Animal and human studies highlight the importance of exposure to a 24-h light-dark cycle in the early postnatal period, for supporting health and wellbeing later in life (Ciarleglio et al., 2011). For example, animal experiments have shown that perinatal light exposure has enduring effects on the physiological and behavioral phenotype in adulthood (Pyter and Nelson, 2006; Weil et al., 2006; Jameson et al., 2023). Likewise, in human studies, season of birth and day length at birth, both proxies for perinatal light exposure, are correlated with the development of several types of cancer (Basta et al., 2010; Makino et al., 2011), pulmonary fibrosis (Hannu et al., 2007), metabolic disorders (Grover et al., 2004; Vaiserman et al., 2009), cardiovascular disease (Lawlor, 2004) and depression (Devore et al., 2018) later in life. According to the developmental origins of the health and disease hypothesis (DOHaD) epigenetic adaptations are made to the fetal/neonatal DNA in response to environmental influences (Langley-Evans and McMullen, 2010; Lacagnina, 2020). Preterm infants are therefore a patient population of particular interest when investigating light exposure since they are more vulnerable to environmental factors considering their immature and developing brain and high risk of complications.

Furthermore, multiple studies have investigated the effect of cycled light in the NICU compared to continuous light or constant darkness on clinical outcomes (Ritchie et al., 2015; Morag and Ohlsson, 2016; Hazelhoff et al., 2021). One recent prospective, randomized multicenter clinical trial reported earlier weight gain and a reduction in length of hospital stay in infants exposed to a light-dark setting (normal room light from 07:00 to 19:00 and darkness (25 lux) from 19:00 to 07:00) compared to the control group (normal room light conditions 24 h per day: 275.82 ± 14 lux during the day and 145.28 ± 14 lux at night) (Sánchez-Sánchez et al., 2022). Differences found between morning and evening melatonin levels in infants exposed to cycled light but not in infants exposed to continuous light (Vásquez-Ruiz et al., 2014; Sánchez-Sánchez et al., 2022) suggest that introducing a light-dark cycle promotes circadian entrainment, already at a very early age.

Which characteristics of light exactly modulate the effects of early-life light exposure on clinical outcomes is currently unknown. This is partly caused by the lack of standardized reporting of light conditions in clinical settings, resulting in heterogeneity and limited technical detail across different studies (Hazelhoff et al., 2021). Useful variables to describe 24-h light-dark cycles in a clinical setting, and more specifically the NICU, are currently lacking. This is challenging, as the NICU lighting environment is inherently difficult to control as the position of the incubators in relation to windows is usually fixed, planned and unplanned care activities require bright light at any time of the day, and high bilirubin levels are treated with the use of intensive phototherapy.

Therefore, in order to understand the interplay between light exposure and clinical outcomes in neonates in the NICU, there is a need to precisely describe the lighting environment and characterize the variation within and between patients in a clinical setting. Using a large retrospectively collected dataset containing bedside ambient light exposure in a Dutch NICU (The Wilhelmina Children’s Hospital NICU in Utrecht, the Netherlands), the goal of this study was to 1) characterize bedside 24-h patterns in light exposure in a NICU setting; 2) explore the use of various metrics to summarize the timing and pattern of 24-h light exposure patterns; and 3) study the association between these metrics and environmental and individual patient characteristics (including season, phototherapy, window proximity and illness severity as measured by the CRIB II score). We hypothesize that infants with a higher illness severity would undergo more clinical interventions characterized by more light exposure and as a consequence would show a more fragmented 24-h light-dark cycle. In our analyses, we focused specifically on the use of rhythmicity metrics that are commonly used to characterize 24-h patterns in human rest-activity cycles (e.g., relative amplitude, interdaily stability, intradaily variability) (Witting et al., 1990; Gonçalves et al., 2015). These metrics provide insight into the prominence, fragmentation, and stability of the 24-h rhythm of the variable of interest (i.e., light exposure in this case) and are of interest as they offer a way to more comprehensively report 24-h patterns in light exposure per patient in studies into the effect of light-dark cycles in clinical settings. As such, our study provides practical considerations and recommendations for future studies into light-dark cycles in the NICU and may be extended to other clinical settings and patient populations.

## 2 Materials and methods

### 2.1 Study population

All very preterm infants (gestational age between 24–32 weeks) who were admitted to the NICU of the Wilhelmina Children’s Hospital (WKZ) in Utrecht, the Netherlands between June 2018 and March 2020 and who stayed in an incubator with a built-in light sensor (Babyleo^®^ TN500, Drager, Germany) were considered for inclusion in this retrospective cohort study. Exclusion criteria were: 1) gestational age at birth above 32 weeks; 2) NICU stay shorter than 5 days; and 3) less than five full days of light measurements recorded at one incubator location after removal of days with more than 120 min of missing data. The threshold of 120 min of missing data was used to avoid imputation of more than 8% of values per day for rhythmicity analysis (see below). The retrospective use of clinically obtained data for scientific inquiries was approved by the Institutional Review Board (IRB) of the University Medical Center Utrecht, the Netherlands (Research protocol nr. 21-066C). The requirement to obtain informed consent from the parents for study participation was waived by the IRB.

### 2.2 Data collection and processing

#### 2.2.1 Patient characteristics

Gestational age at birth, birth weight, incubator location, phototherapy exposure, the month of birth, and location of the incubator were obtained from medical records. The month of birth was categorized into autumn (Sept, Oct, Nov), winter (Dec, Jan, Feb), spring (Mar, Apr, May), and summer (Jun, Jul, Aug). The location of the incubator within the open-bay NICU was categorized into three locations depending on their distance from the nearest window, which is dictated by the specific layout of our NICU, with one row consisting of five incubators directly adjacent to the window (‘window location’), a second row of two incubators and approximately 6 m from the window (‘in-room location’), and incubators in a more secluded, separate environment (‘secluded location’). The position of incubators and their directionality toward windows is visualized in a detailed NICU map (Supplementary Figure S1). In addition, the clinical risk index for babies II (CRIB-II), a validated measure of initial mortality risk and illness severity in preterm infants born between the gestational age of 22–32 weeks, was calculated based on variables obtained from medical records (birth weight, body temperature, and base excess within the first 12 h of life next to gestational age and sex), as described in Ezz-Eldin et al. (Ezz- Eldin et al., 2015).

#### 2.2.2 Continuous incubator light measurements

Light levels (illuminances in lux) were recorded at 1-min intervals by a built-in light sensor in the incubator (Babyleo^®^ TN500, Dräger, Germany). The sensor had a measurement range from 3 to 999 lux (Dräger, 2019) and was positioned at the head side of the baby, at eye level, and measured on a vertical plane. We compared the incubator light sensor to an industry-standard light meter (SDL400 Light Meter/Datalogger, EXTECH, Nashua, United States) (Supplementary Figure S2). A linear relationship between the light exposure values returned by the incubator sensor and the validated light meter were found, with the incubator sensor overestimating the actual light exposure values by 28%, possibly due to its spatial arrangement in the incubator. Throughout the study, light levels were recorded every minute during the entire time the infant was in the incubator, amounting to 1,440 measurements per day. All data from the first full day (from midnight to midnight) until the last full day that an infant stayed in the incubator were included in further analyses. Light measurements recorded during phototherapy were set to 3 lux (the lower limit of the light sensor), as the infant’s eyes are covered during this time with an eye protector (Biliband^®^ Eye Protector for Newborn Phototherapy, Natus Medical Incorporated, San Carlos, California, United States) that blocks close to 99% of the light (Natus, 2012).

### 2.3 Data processing and analysis

#### 2.3.1 Data processing

All data processing, visualization, and analysis was performed using R (version 4.0.3) and relevant Tidyverse packages (version 1.3.2) (Wickham et al., 2019).

#### 2.3.2 Characterization of 24-h light exposure patterns

To characterize the 24-h patterns of light exposure that infants received in the NICU, various metrics were calculated from the continuous light measurements. First, to quantify 24-h light exposure, we computed 1) Average total light exposure per nurse shift comprising morning (from 07:30–15:30), afternoon (from 15:30–23:15), and night (from 23:15–07:30), by calculating the mean of available light recordings in the dataset during those time windows; 2) the overall light exposure over the 24-h period by calculating the percentage of light exposure within different ranges (<5 lux, 5–20 lux, 20–50 lux, and >50 lux) for each 1-min time bin between midnight and midnight; and 3) the time above threshold (TaT), calculated as the average time in hours per day that an infant was exposed to light exposure above a certain threshold value–we explored thresholds between 1 and 100 lux.

Secondly, to characterize the 24-h rhythmicity in light exposure, we calculated 1) interdaily stability (IS), 2) intradaily variability IV), 3) relative amplitude (RA), and 4) the start time (in 24-h clock time) of the darkest 5 h (D5) and brightest 10 h (B10) in 24-h clock time as described elsewhere (Van Someren et al., 1999). IS reflects the consistency of a 24-h pattern over consecutive days, ranging from 0 for a complete lack of consistency to 1 for complete day-to-day similarity. IV is a measure of fragmentation, with values ranging from 0 for low fragmentation and 2 for high fragmentation. Finally, RA represents the contrast between the darkest 5 h and the brightest 10 h, ranging from 0 to 1. To compute these metrics, illuminance values were log-transformed and missing data points were imputed with a random number generated from a normal distribution with a mean and standard deviation of the light recordings from the 15 min preceding and following the missing data points. Given the inclusion criteria described above (days with more than 120 min of missing data are excluded from analysis), a maximum of 120 min per day were missing and thus imputed. Subsequently, the different rhythmicity metrics were computed using the R package nparACT (version 0.8) (Blume et al., 2016). For IS, IV, and RA, individual patient values, medians, and interquartile ranges were plotted to visualize the variability of these metrics across patients. For the timing of D5 and B10, individual patient values, circular means, and the mean resultant vector length (a measure of the spread of circular data, ranging between 0 and 1) were calculated using the R package circular (version 0.4-95) (Agostinelli and Lund, 2022). We also calculated the Light Regularity Index (LRI) as described by Hand et al. (2023), based on the formula reported by Lunsford-Avery et al. (2021). The Light Regularity Index is calculated based on the probability that an individual is in the same state (above vs. below a threshold of light exposure in lux) at any two time points, 24 h apart. We calculated LRI for three different thresholds (≥10, ≥20, and ≥50 lux). LRI is 0 when the 24-h light exposure is completely randomly distributed and 100 when light exposure is always in the same state at each time point 24 h apart.

#### 2.3.3 Statistical analysis

Patient characteristics were summarized either as median and the interquartile range (IQR), or as counts and percentages. Average light levels per nurse shift (morning shifts from 07:30–15:30; afternoon shifts from 15:30–23:15; and night shifts from 23:15–07:30) were compared using a linear mixed effects model with R package lme4 (version 1.1-29) and lmerTest (version 3.1-3) with patientID included as random effect (Bates et al., 2015; Kuznetsova et al., 2017). Posthoc pairwise comparisons between shifts were obtained using the R package emmeans, using Tukey method to adjust *p*-values for multiple testing (version 1.7.0) (Lenth R, 2021).

The effects of season and severity score on relative amplitude, interdaily stability and intradaily variability were tested by means of an ANOVA, with Tukey *post hoc* testing. A Kruskal–Wallis test was used in case residuals of the ANOVA were not normally distributed (*p*-value of Shapiro-Wilk test <0.05). The effects of phototherapy and bed location on relative amplitude, interdaily stability an intradaily variability were tested with a *t*-test. A Wilcoxon Sign tests was used in case residuals of the *t*-test were not normally distributed (*p*-value of Shapiro-Wilk test <0.05). A *p*-value less than 0.05 was considered to be significant.

## 3 Results

### 3.1 Patient characteristics

A flowchart of patient selection can be found in Supplementary Figure S3. In total, sufficient light recordings were available from 79 infants. The median length of NICU admission was 43 days (IQR: 24-73). Mortality during NICU stay was 10%. In total, 87% of infants (n = 69) received phototherapy while they stayed in the incubator with the built-in light sensor. In those 69 infants, phototherapy was given during 30% (median, IQR: 19%–40%) of the total duration of the light recordings. The majority of infants (76%) had a CRIB-II score between level two and three. Most infants (75%) stayed in incubators placed next to the window. Patient characteristics of the study population are further described in Table 1.

**TABLE 1:** Patient characteristics.

Characteristic	N = 79
**Sex**	
Female	36 (46%)
Male	43 (54%)
**Gestational age, weeks**	27 (26–29)
**Birth weight, grams**	920 (770–1,135)
**Season of birth**	
Winter	20 (25%)
Spring	18 (23%)
Summer	20 (25%)
Autumn	21 (27%)
**Deceased**	8 (10%)
**Phototherapy during NICU stay**	69 (87%)
**NICU stay, days**	43 (24–73)
**Bed location**	
Window location	20 (25%)
In-room location	59 (75%)
Secluded location	0 (0%)
**CRIB-II level**	
Level 1	8 (10%)
Level 2	32 (41%)
Level 3	28 (35%)
Level 4	4 (5.1%)
Unknown	7 (8.9%)

Shown are number N) (%) or median (interquartile range). NICU: neonatal intensive care unit; CRIB-II: clinical risk index for babies II.

### 3.2 Quantification of 24-h light exposure

**FIGURE 1:**
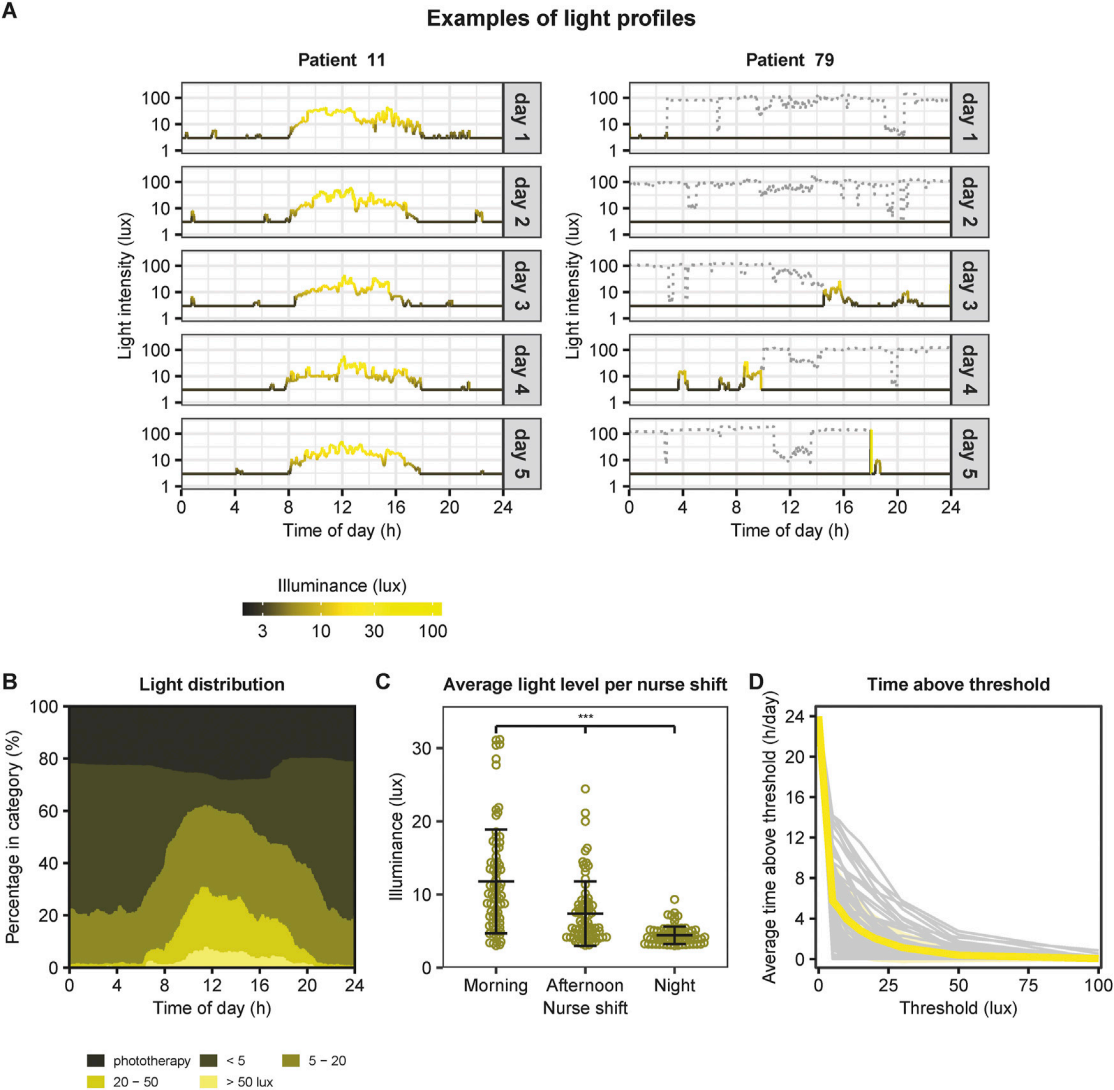
Overview of 24-h light exposure in the NICU. **(A)** Examples of light profiles. On the left an example of a patient with a clear 24-h light-dark cycle. This light profile illustrates the light exposure in lux (logarithmic scale) over 24 h. Grey boxes indicate the day with a total of 5 days. On the right an example of a patient without a clear 24-h light-dark cycle due to phototherapy. The grey dotted lines indicate light intensities measured during phototherapy. As the infants’ eyes were covered during phototherapy, values were set to 3 lux for further analyses (indicated by the black lines below the grey dotted lines). **(B)** Average light distribution over 24-h across all NICU days and patients (N = 79), subdivided in light exposure categories (<5 lux, 5–20 lux, 20–50 lux, and >50 lux) and presented as percentages. **(C)** Average light exposure per nurse shift comprising morning (from 07:30–15:30), afternoon (from 15:30–23:15) and night (from 23:15–07:30). ***: *p* < 0.001 (posthoc comparisons following linear mixed effects model with patientID as random effect). Data points represent the mean light exposure per patient across multiple days. Horizontal lines and error bars indicate the mean and standard deviation of each nurse shift. **(D)** Average time in hours per day above a certain light exposure threshold. Grey lines indicate the individual patients, and the yellow line illustrates the mean.

In total we analyzed a dataset containing 1,628,239 light measurements, collected over 1,131 days. Per infant, 13 [10–15; median, IQR] days of data were available, with a range of 5–44 days. Average light exposure per patient was 7.82 ± 3.93 lux (mean ± SD). We observed a large degree of variability in light exposure between patients as well as between different days within one patient, especially due to phototherapy (Figure 1A). Figure 1B illustrates the average light distribution over 24 h across all NICU days and patients. Measurements of higher light intensity (20–50 lux and >50 lux) clustered during the day, and of lower intensity (below 20 lux) clustered during the night, in line with the presence of a 24-h light cycle. There were significant differences in light exposure between all the three shifts on a day (linear mixed effects model, F (2, 156) = 89.7, *p* < 0.001). We found an average light exposure of 11.8 ± 7.09 lux (mean ± SD) in the morning, 7.38 ± 4.40 lux in the afternoon and 4.41 ± 1.19 lux in the night (Figure 1C). As can be seen in Figure 1D light exposure was generally low, with minimal time above 75 lux. Also the Light Regularity Index (LRI) was highly variable between patients (Supplementary Figure S4). An increase in the light threshold resulted in a higher LRI (≥10 lux: 68.9 ± 17.8 (mean ± SD); ≥20 lux: 77.7 ± 16.7; ≥50 lux: 91.8 ± 10.4).

### 3.3 Characterization of 24-h rhythmicity in light exposure

**FIGURE 2:**
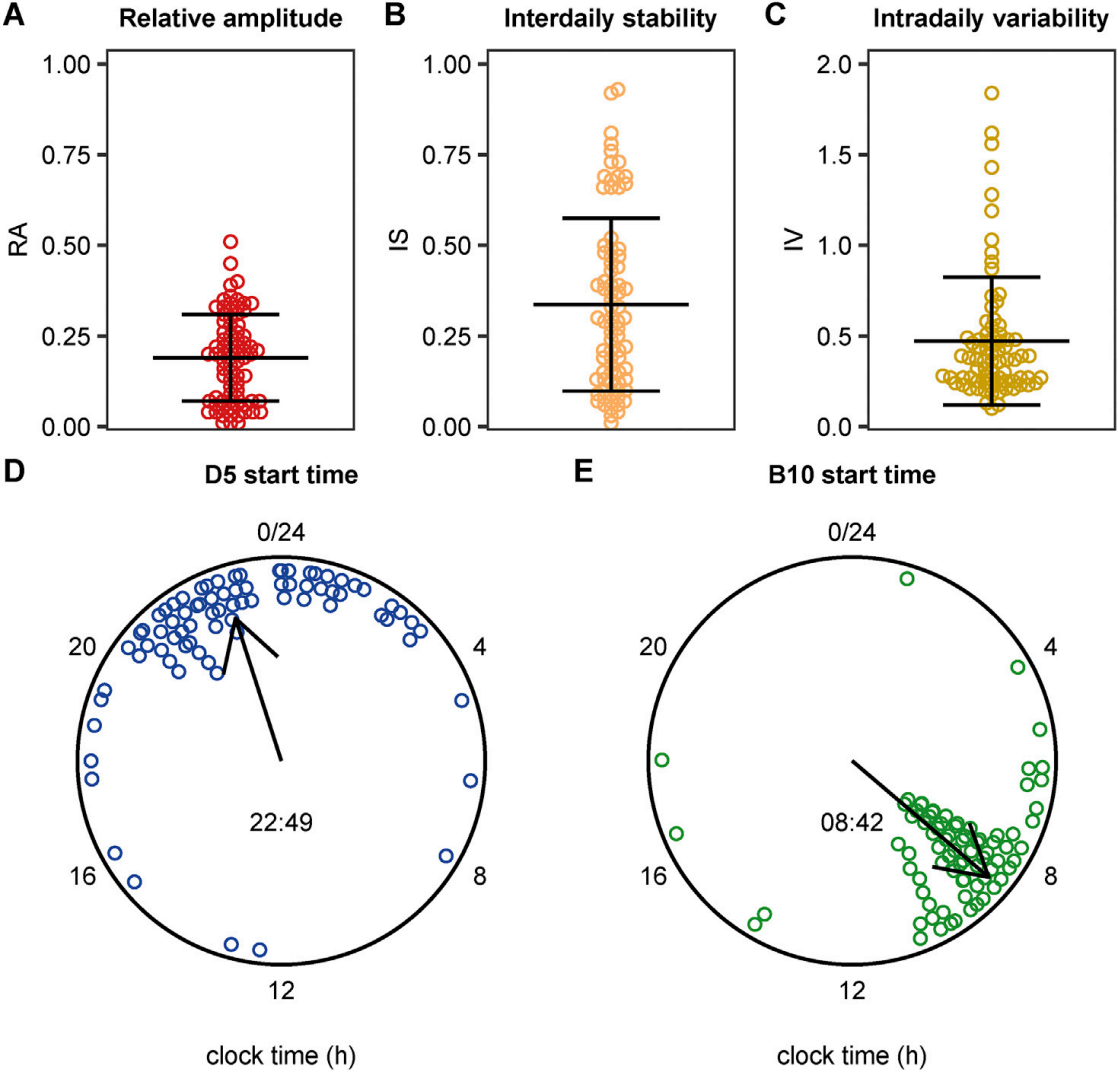
Distribution of light exposure patterns including the individual values. **(A)** Boxplot indicating the median relative amplitude (RA) with the interquartile range (IQR). RA ranges from 0 (no contrast between the darkest 5 h and the brightest 10 h) and 1 (maximum contrast between the darkest 5 h and the brightest 10 h). **(B)** Boxplot indicating the median interdaily stability (IS) with the IQR. IS ranges from 0 for a complete lack of consistency between days to 1 for complete day-to-day similarity **(C)** Boxplot indicating the median intradaily variability (IV) with the IQR. IV ranges from 0 for low fragmentation and 2 for high fragmentation. **(D, E)** Circular plot showing the start time of the 5 darkest hours (D5) (panel D) and of the brightest 10 h (B10) (panel E). Data points represent individual patients, the direction of the arrow represents the circular mean across patients, and its length represents the mean resultant length (a measure of the spread of circular data).

Prior to rhythmicity analysis, missing light measurements were imputed. In total, this concerned 1,841 data points (0.11% of all data points). Results showed indications of a 24-rhythmicity, albeit weak: we found a low relative amplitude of 0.19 ± 0.12 (mean ±0.12), a quite low mean interdaily stability of 0.34 ± 0.24, and a mean intradaily variability of 0.47 ± 0.35 (Figures 2A–C). The timing of the darkest 5 h (D5) started on average at 22:47 and the 10 brightest hours (B10) started on average at 08:48 (Figures 2D,E). As visualized in Figure 2, considerable variation was observed in these metrics between patients, which prompted us to investigate the effect of environmental and individual patient characteristics on these metrics.

### 3.4 Factors influencing light exposure in the NICU

**FIGURE 3:**
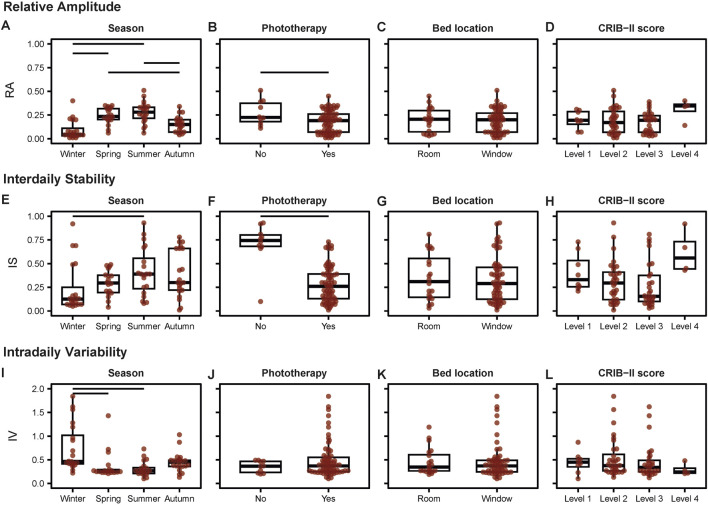
Associations of **(A–D)** relative amplitude, **(E–H)** interdaily stability, and **(I–L)** intradaily variability with environmental and individual patient characteristics. Horizontal lines indicate significant (*p* < 0.05) posthoc comparisons in case main effects were significant. See Supplementary Table S2 and Supplementary Table S1 for full details and statistics.

We next explored the association between light rhythmicity metrics and various environmental and individual patient characteristics, including season, phototherapy, bed location, as well as illness severity. Results of the main effects are summarized in Supplementary Table S1 and between-group comparisons are visualized in Figure 3 and summarized in Supplementary Table S2 **.** A significant main effect of season was found on relative amplitude, interdaily stability, and intradaily variability and of phototherapy on relative amplitude and interdaily stability, but not on intradaily variability (Supplementary Table S1). Specifically, *post hoc* tests of the significant main effects revealed that relative amplitude was higher during spring and summer compared to winter and autumn (Figure 3A). Furthermore, interdaily stability was higher during summer compared to winter (Figure 3E). Intradaily variability was found to be highest in the winter compared to spring and summer (Figure 3I). Moreover, relative amplitude and interdaily stability were higher in patients that did not receive phototherapy during their stay compared to those who did (Figures 3B,F). In addition, a significant main effect was found of illness severity on interdaily stability (Supplementary Table S1), but no posthoc comparisons were significant (Supplementary Table S2). Lastly, we found no significant main effects of illness severity on relative amplitude or intradaily variability and of bed location (next to the window or ∼6 m away from the window) on any of the variables (Supplementary Table S1 and Figure 3)**.**

## 4 Discussion

The aim of this study was to capture the variability in 24-h light-dark cycles that infants in the NICU are exposed to and to investigate the association between these variables and various environmental and individual patient characteristics. Our results show a 24-h light-dark cycle was present in the NICU with significant differences in light exposure between the three different nurse shifts, with the highest values in the morning and the lowest values at around midnight. However, light exposure was generally low, with illuminances rarely surpassing 75 lux, and highly variable between patients and across days within in a single patient. Furthermore, the season of birth (and consequently, of the infants’ NICU stay) and phototherapy were revealed as important factors that influence 24-h light-dark cycles, whereas no effect of bed location and illness severity were observed. Overall, this study provides a step towards more detailed and individualized reporting of light exposure in clinical settings.

In general, the light levels observed in our study were generally low across the 24-h period. This is in line with previous studies that characterized light levels in different hospital settings, in which median daytime light levels ranged between 40–150 lux (Durrington et al., 2017; Fan et al., 2017; Greenfield et al., 2020; Lusczek and Knauert, 2021). In addition, in a study comparing two different NICU designs, it was found that daytime light levels were lower in an open ward design (comparable to the NICU in our study) compared to in pods and single-family rooms (69 vs. 368 lux) (Aita et al., 2021).

Despite the generally low light levels, a 24-h light-dark cycle was present in the NICU with significant differences in light exposure between the three different nurse shifts. This finding was unexpected since other studies and clinical practice present the NICU as an environment where clear environmental 24-h rhythms are usually absent, exposing the infant to irregular or continuous illumination (McKenna and Reiss, 2018; Hazelhoff et al., 2021). However, the optimal contrast in daytime and nighttime light levels in terms of neonatal development remains to be established. Moreover, in intervention studies that investigate the effect of a light-dark cycle in the NICU on clinical outcomes, the control condition is often described as either constant light or constant near-darkness throughout the 24-h period (Morag and Ohlsson, 2016), without a detailed description of the light to which patients are exposed. Our study shows that even in an anticipated near-darkness NICU environment without an official set lighting regime, a 24-h light-dark cycle can be present. This highlights the need for more fine-grained characterization of light exposure in clinical trials that investigate the effect of light interventions.

To characterize a 24-h light-dark cycle per patient we explored different rhythmicity metrics, including relative amplitude, interdaily stability, intradaily variability, and timing of the darkest 5 h, and the brightest 10 h. These variables have been traditionally used to describe 24-h rhythms in actigraphy data to characterize human rest-activity cycles (Winkler et al., 2005; Carvalho-Bos et al., 2007; Paudel et al., 2011) but can also be used to describe light data (Martinez-Nicolas et al., 2014). In our study, we found modest values for the relative amplitude, interdaily stability and intradaily variability, which indicates 1) a medium contrast between the darkest 5 h and the brightest 10 h over 24 h, 2) a moderate consistency of a 24-h light-dark cycle across days and 3) limited fragmentation of the 24-h light-dark cycle, with a high degree of variability among infants. Regularity of light exposure was assessed using the LRI developed by Hand et al. (2023) and also showed large interindividual differences. Given the variability between infants, these rhythmicity metrics may prove to be useful in characterizing 24-h light-dark cycles in NICUs, with possible extension to hospitals in general, and adopted by studies that investigate the effect of light-dark cycles to health outcomes in clinical settings. In general, the development of standardized metrics to characterize light exposure in clinical settings would facilitate the comparison of different studies and the interpretation of the results, which are eventually crucial for providing recommendations on the optimal light exposure in NICUs. Therefore, also in clinical settings, the development of a standardized framework for light dosimetry studies could be of great value (Hartmeyer et al., 2022).

We next explored the effect of various environmental and individual patient characteristics on light rhythmicity metrics in order to determine to what extent they contributed to the variability observed across patients. Firstly, a clear effect of season of birth was found. The 24-h light-dark cycle to which infants born in summer were exposed was characterized by a higher relative amplitude and interdaily stability and a lower intradaily variability (both indicative of stronger rhythmcity) compared to those born in winter. This indicates that infants born in the summer season and admitted to the NICU are exposed to a more robust 24-h light-dark cycle compared to infants born in the winter season, suggesting that, in our NICU, outdoor light conditions influence the indoor environment. In this context, it is interesting to note that different incubator locations (directly adjacent to the window vs. ∼6 m from the window) did not significantly influence light exposure in the NICU. This is in contrast to a study reporting on light levels in adult intensive care units, in which significantly higher light levels were found near beds located next to a window compared to beds located in rooms without a window (Durrington et al., 2017). In this study, the infants (and light sensors) situated in the “window location incubators” are faced away from the windows. In contrast, the infants (and light sensors) situated in the “in-room incubators” approximately 6 m from the window are directed towards the windows. This could at least partly explain why we found no significant effect of bed location on any of the light rhythmicity metrics. Furthermore, we hypothesized that infants with a higher illness severity would undergo more clinical interventions characterized by more light exposure and as a consequence would show a more fragmented 24-h light-dark cycle. However, no impact of illness severity on 24-h light-dark cycles was seen. However, this finding should be interpreted with caution since there was a limited number of infants with a very low (level 1) and very high (level 4) CRIB II illness severity score. Additionally, 87% of infants were exposed to phototherapy during their NICU stay, during which their eyes are covered with an eye protector. Unsurprisingly, phototherapy exposure was associated with reduced relative amplitude and interdaily stability. The large proportion of infants that receive phototherapy in the NICU, and the considerable duration of phototherapy exposure per infant (30% of their total duration of light recordings), raise the question how to control for the influence of phototherapy in future light intervention studies in the NICU.

There are several limitations of this study worth mentioning. We made use of the built-in light sensor of the infants’ incubator, which we compared against a validated photometer. Although the relationship between the built-in light sensor and the photometer was linear across the range of observed light exposure values, the light sensor tended to consistently overestimate the values. Another limitation includes the light sensor in the incubator measuring light exposure on a vertical plane, while measuring in a horizontal plane, in the viewing direction of the infants would be more ideal. Furthermore, during times of phototherapy exposure, light exposure was set to 3 lux (the minimum value of the built-in light sensor), based on the assumption that the eye protectors prevented light from reaching the eyes during these times. In practice, it is possible that occasionally some light may have entered via the sides of the protector, but we expect the effect of this to be minimal. One of the strengths of this study is the continuous patient-level measurement of light. A next step would be to also consider the spectral properties of the light. For future studies into light in clinical settings, it is important to more precisely report light exposure. For example, intervention studies that investigate the effect of cycled light in the NICU would benefit from more detailed characterization of light patterns for both reproducibility purposes and to explore the properties of light that contribute to the observed clinical effects.

In conclusion, we provide a detailed characterization of patient-level 24-h light-dark cycles in a neonatal intensive care unit. We observe large variability of light exposure between patients and between different days within a single patient. Our results show that season of birth and phototherapy exposure have a significant impact on the different light variables, whereas bed location and illness severity levels did not. As such, this study provides insight into the environmental and individual patient characteristics that affect light exposure in a clinical setting and may help future intervention studies into cycled light to more completely report 24-h light exposure patterns. This may lead to a better understanding of the effects of light-dark cycles in the NICU on clinical outcomes in preterm infants. Eventually, this may provide input for new NICU designs, possibly changing it into environments that strengthen the development of circadian rhythms in preterm infants and in turn could contribute to the improvement of health in this vulnerable patient population.

## Data Availability

The datasets for this article are not publicly available due to concerns regarding patient anonymity. Requests to access the datasets should be directed to the corresponding author (JD). Any data sharing will be subject to meeting the Privacy Regulations of UMC Utrecht, the General Data Protection Regulation (GDPR) and the General Data Protection Regulation Implementation Act. Requests to access the datasets should be directed to J.Dudink@umcutrecht.nl.
