# Supplementary material for: Characterizing light-dark cycles in the Neonatal Intensive Care Unit: a retrospective observational study
Source: Front Physiol. 2023 Aug 16;14:1217660. doi: 10.3389/fphys.2023.1217660 (PMC10469299; doi:10.3389/fphys.2023.1217660)
Supplement: Supplementary file 1 [file DataSheet1.pdf]

Supplemental Material

Characterizing light-dark cycles in the Neonatal Intensive Care Unit: a retrospective observational study

Isabelle A. van der Linden[1†], Esther M. Hazelhoff[2†], Eline R. de Groot[1], Daniel C. Vijlbrief[1], Luc J.M. Schlangen[3], Yvonne A.W. de Kort[3], Marijn J. Vermeulen[4], Demy van Gilst[4], Jeroen Dudink[1^*], Laura Kervezee[2^*]

†These authors have contributed equally to this work and share first authorship
^These authors share last authorship

[1]Department of Neonatology, Wilhelmina Children's Hospital, University Medical Center Utrecht, Utrecht, Netherlands
[2]Laboratory for Neurophysiology, Department of Cellular and Chemical Biology, Leiden University Medical Center, Leiden, Netherlands
[3]Department of Industrial Engineering and Innovation Sciences, Eindhoven University of Technology, Eindhoven, Netherlands
[4]Department of Neonatal and Pediatric Intensive Care, Division of Neonatology, Erasmus MC - Sophia Children's Hospital, Rotterdam, Netherlands

*** Correspondence:**
Corresponding Authors:
Laura Kervezee (L.Kervezee@lumc.nl); Jeroen Dudink (J.Dudink@umcutrecht.nl)

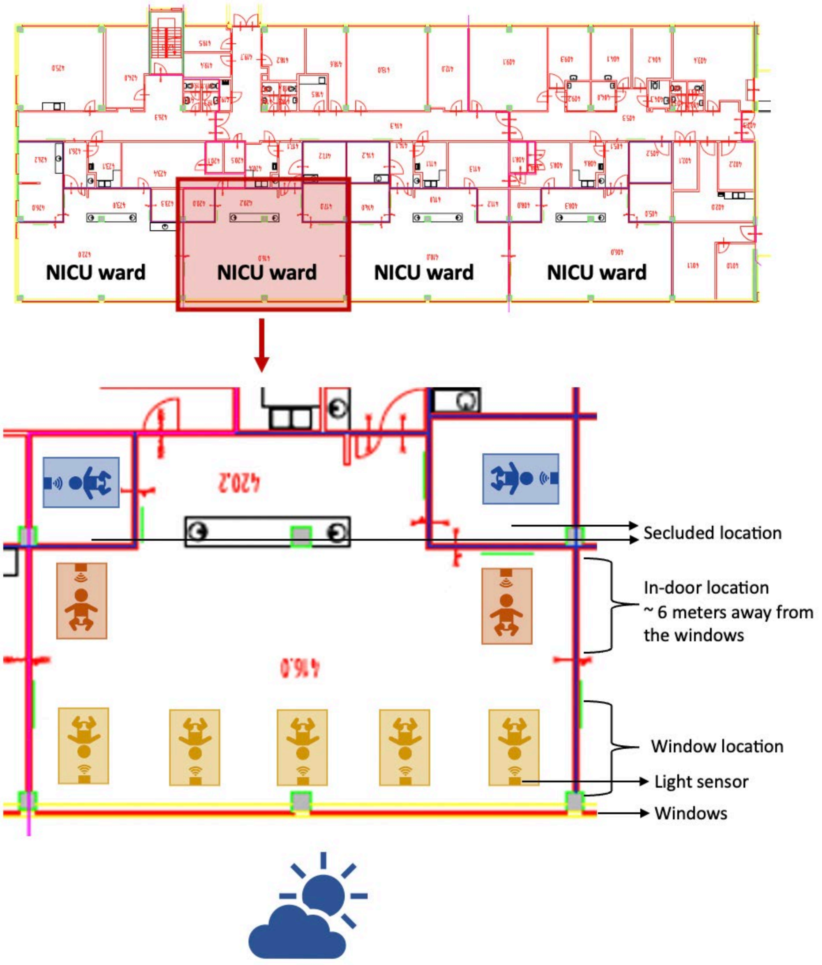

Supplemental Figure 1. Map of incubator locations within the open-bay NICU. The scaled map on top shows the wing consisting of four NICU wards. Below a detailed graphical representation of one NICU ward is displayed. The location of the incubator is categorized into three locations depending on their distance from the nearest window. One row consisting of five incubators (colored yellow) directly adjacent to the window ('window location'), a second row of two incubators (colored red) and approximately 6 meters from the window ('in-room location'), and incubators (colored blue) in a more secluded, separate environment ('secluded location').

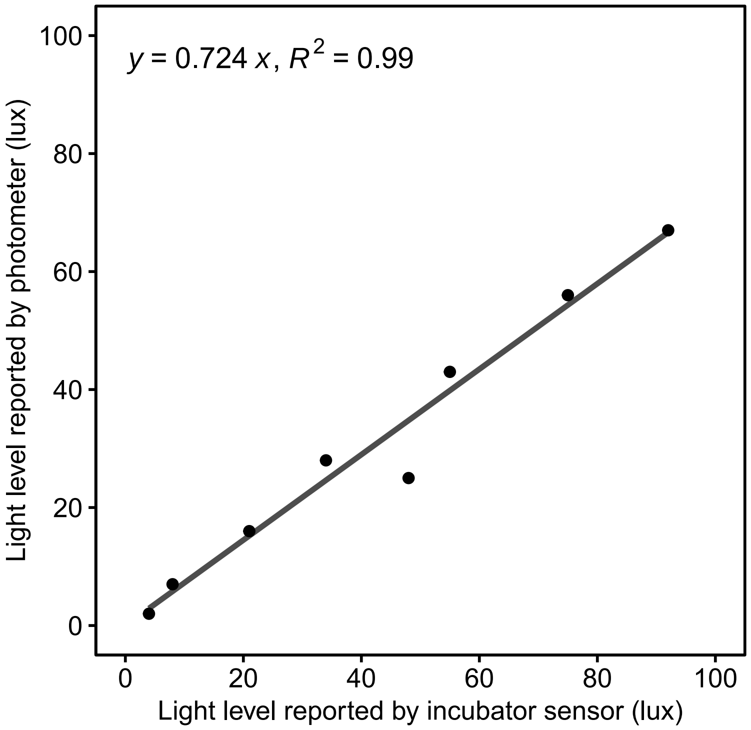

Supplemental Figure 2. Comparison of incubator light sensor with photometer. Light exposure (illuminance in lux) as measured by the light sensor in the incubator (Babyleo® TN500, number: 341650, Kpl F343 year 2018, Dräger, Germany) versus light exposure measured by a photometer (SDL400: Light Meter/Datalogger, EXTECH, Nashua, United States).

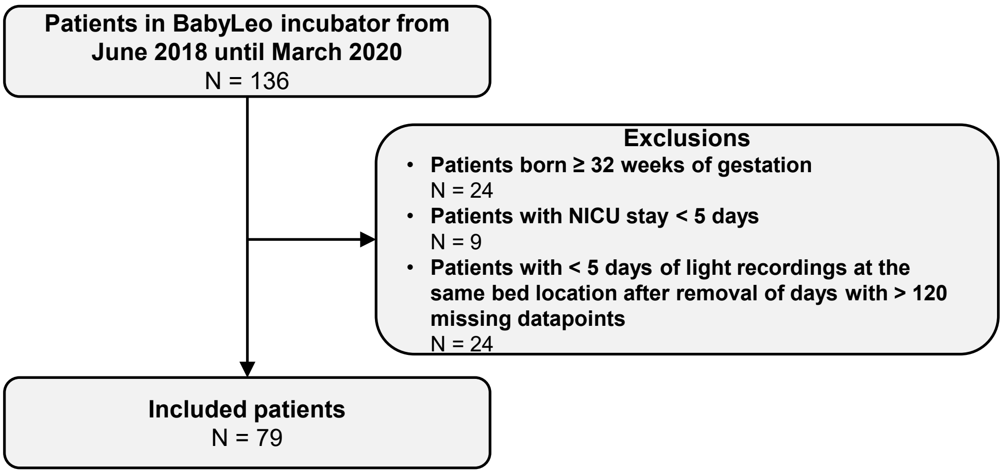

Supplemental Figure 3. Flowchart of patient selection.

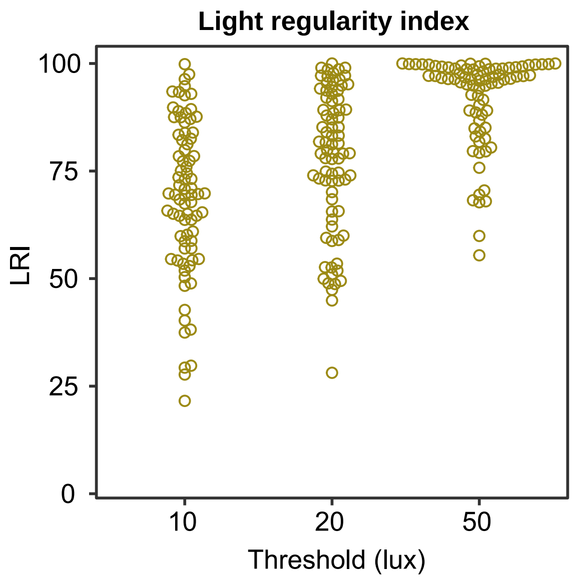

Supplemental Figure 4. Light regularity index (LRI) calculated using ≥10, ≥20, and ≥50 lux as thresholds. Data points represent individual patients. Mean and standard deviations per threshold are reported in the main manuscript text. LRI is 0 when the 24-hour light exposure is completely randomly distributed and 100 when light exposure is always in the same state at each time point 24 hours apart.

Supplemental Table 1. Main effects of the associations between light rhythmicity metrics and environmental and individual patient characteristics.

		Test statistic#	P-value
**Season of birth**			
	Relative amplitude	F(3, 75) = 14.9	< 0.0001
	Interdaily stability	H(3) = 10.9	0.0246
	Intradaily variability	H(3) = 20.5	0.00013
**Phototherapy (yes vs no)**			
	Relative amplitude	H(1) = 3.97	0.0464
	Interdaily stability	H(1) = 17.0	< 0.0001
	Intradaily variability	H(1) = 0.523	0.470
**Bed location (window vs in-room)**			
	Relative amplitude	H(1) = 0.073	0.787
	Interdaily stability	H(1) = 0.076	0.782
	Intradaily variability	H(1) = 0.031	0.861
**CRIB-II Score**			
	Relative amplitude	H(3) = 4.80	0.187
	Interdaily stability	H(3) = 8.93	0.0302
	Intradaily variability	H(3) = 2.94	0.401

CRIB-II: clinical risk index for babies II.

Supplemental Table 2. Post-hoc comparisons of significant main effects of the associations between light rhythmicity metrics and environmental and individual patient characteristics.

	P-values		
	**Relative amplitude**	**Interdaily stability**	**Intradaily variability**
**Season of birth**	Tukey-HSD	Dunn test	Dunn test
Autumn - Spring	0.0316	0.834	0.094
Autumn - Summer	< 0.0001	0.834	0.071
Autumn - Winter	0.189	0.070	0.234
Spring - Summer	0.652	0.720	0.878
Spring - Winter	< 0.0001	0.474	0.001
Summer - Winter	< 0.0001	0.030	0.0006
**CRIB-II Score**	-	Dunn test	-
Level 1 - Level 2	ND	0.678	ND
Level 1 - Level 3	ND	0.271	ND
Level 1 - Level 4	ND	0.678	ND
Level 2 - Level 3	ND	0.678	ND
Level 2 - Level 4	ND	0.148	ND
Level 3 - Level 4	ND	0.051	ND

ND: not determined because main effect was not significant. CRIB-II: clinical risk index for babies II. HSD: honestly significant difference.
